# Supplementary material for: Redundant roles of EGFR ligands in the ERK activation waves during collective cell migration
Source: Life Sci Alliance. 2021 Oct 19;5(1):e202101206. doi: 10.26508/lsa.202101206 (PMC8548211; doi:10.26508/lsa.202101206)
Supplement: Supplementary file 6 [file LSA-2021-01206_Supplemental_Data_1.docx]

Supplemental Data 1

EKARrEV-NLS

YPet, WW domain, EV linker, ERK substrate peptide, mTurquoise, NLS

ATGGTGAGCAAGGGCGAGGAGCTGTTCACCGGGGTGGTGCCCATCCTGGTCGAGCTGGACGGCGACGTAAACGGCCACAAGTTCAGCGTGTCCGGCGAGGGCGAGGGCGATGCCACCTACGGCAAGCTGACCCTGAAGCTTCTATGCACCACCGGCAAGCTGCCCGTGCCCTGGCCCACCCTCGTGACCACCCTGGGCTACGGCCTGCAGTGCTTCGCCCGCTACCCCGACCACATGAAGCAGCACGACTTCTTCAAGTCCGCCATGCCCGAAGGCTACGTCCAGGAGCGCACCATCTTCTTCAAGGACGACGGCAACTACAAGACCCGCGCCGAGGTGAAGTTCGAGGGCGACACCCTGGTGAACCGCATCGAGCTGAAGGGCATCGACTTCAAGGAGGACGGCAACATCCTGGGGCACAAGCTGGAGTACAACTACAACAGCCACAACGTCTATATCACCGCCGACAAGCAGAAGAACGGCATCAAGGCCAACTTCAAGATCCGCCACAACATCGAGGACGGCGGCGTGCAGCTCGCCGACCACTACCAGCAGAACACCCCCATCGGCGACGGCCCCGTGCTGCTGCCCGACAACCACTACCTGAGCTACCAGTCCGCCCTGTTCAAAGACCCCAACGAGAAGCGCGATCACATGGTCCTGCTGGAGTTCCTGACCGCCGCCGGGATCACTGAGGGCATGAACGAGCTGTACCTCGAGATGGCGGACGAGGAGAAGCTGCCGCCCGGCTGGGAGAAGCGCATGAGCCGCAGCTCAGGCCGAGTGTACTACTTCAACCACATCACTAACGCCAGCCAGTGGGAGCGGCCCAGCGGCAACAGCAGCAGTGGTGGCAAAAACGGGCAGGGGGAGCCTGCCAGGGGTACCAGTGCTGGTGGTAGTGCTGGTGGTAGTGCTGGTGGTAGTGCTGGTGGTAGTGCTGGTGGTTCCGGCAGTGCTGGTGGTAGTGCTGGTGGTAGTACCAGTGCTGGTGGTAGTGCTGGTGGTAGTGCTGGTGGTAGTGCTGGTGGTAGTGCTGGTGGTTCCGGCAGTGCTGGTGGTAGTGCTGGTGGTAGTACCAGTGCTGGTGGTAGTGCTGGTGGTAGTGCTGGTGGTAGTGCTGGTGGTAGTGCTGGTGGTTCCGGCAGTGCTGGTGGTAGTGCTGGTGGTAGTACCAGTGCTGGTGGTAGTGCTGGTGGTAGTGCTGGTGGTAGTGCTGGTGGTAGTGCTGGTGGTTTCCGGACTCCTGATGACGCCATGCTACACGGCAAAGCTGTCATTCCAATTTCCGGGCGGCCGCATGGTGAGCAAGGGCGAGGAGCTGTTCACCGGGGTGGTGCCCATCCTGGTCGAGCTGGACGGCGACGTAAACGGCCACAGGTTCAGCGTGTCCGGCGAGGGCGAGGGCGATGCCACCTACGGCAAGCTGACCCTGAAGTTCATCTGCACCACCGGCAAGCTGCCCGTGCCCTGGCCCACCCTCGTGACCACCCTGACCTGGGGCGTGCAGTGCTTCAGCCGCTACCCCGACCACATGAAGCAGCACGACTTCTTCAAGTCCGCCATGCCCGAAGGCTACGTCCAGGAGCGTACCATCTTCTTCAAGGACGACGGCAACTACAAGACCCGCGCCGAGGTGAAGTTCGAGGGCGACACCCTGGTGAACCGCATCGAGCTGAAGGGCATCGGCTTCAAGGAGGACGGCAACATCCTGGGGCACAAGCTAGAGTACAACTACATCAGCCACAACGTCTATATCACCGCCGACAAGCAGAAGAACGGCATCAAGGCCCACTTCAAGATCCGCCACAACATCGAGGACGGCGGCGTGCAGCTCGCCGACCACTACCAGCAGAACACCCCCATCGGCGACGGCCCCGTGCTGCTGCCCGACAACCACTACCTGAGCACCCAGTCCGCCCTGAGCAAAGACCCCAACGAGAAGCGCGATCACATGGTCCTGCTGGAGTTCGTGACCGCCGCCGGGATCACTCTCGGCATGGACGAGCTGTCTAGACCTAAGAAGAAGCGTAAGGTG

Canine EGF

ATGCTGCTCCCCCTTATCATTCTGTGGCCGGTAGTTTTTAAATGTAGTTTTGCTAGTCTCTCAGACCCGGAGAACTGGAACTGTCCTGAAGTCTCTCCCTCAGGAAAGGGGAGCCCTGCTTGTGTGGGTCCTGCACCCTTCTTAATTTTCTCCCATGGAATCAGTATCTTTAGGATTGACCTGGAAGGCACTAATCATGAGCAATTGGTGGCAGATGCTGGTGTATCAGTGATCATGGATTTTCATTATAATAAAGAAAGAATCTATTGGGTAGATCCAGAAAGACAACTTTTGCAAAGAGTTTTTCTGAATGGGACAAGGCAAGAGAGAGTATGCAATATAGAGAAAAATGTTTCAGGAATGGCAATAAATTGGATAAATGAAGAACTTATTTGGTCAAATCAACAGGAAGGAATCATCACAGTAACAGATATGAAAGGAAACAATTCGCGAGTTCTCTTAAGAGCCTTAAACTATCCTGCAAATGTAGCAATTGATCCAATAGAAAGGTTTATATTTTGGTCTTCAGAGGTGGCAGTGGCTGGCAGCCTTCACAGAGCAGATCTCAATGGTGTGGAAGAGAAGATTCTTTTACAGACATCAGAAAGAATAACAGCTGTGTCATTGGATGTGCTTGATAAACAGCTTTTTTGGATTCAGTACAGCAGAGATGGAAGCAATTCTCATATTTATTCCTGTAATTATGATGGAGGTTCTGTCCATCTTAGCAAACATCTTACACAGCATAATTTTTTTGCAATGTCCCTTTTTGGGAATCAGATCTTCTATTCAACATGGAAAAAGAAGACAATTTGGATAGCTAACAAACACAGTGGGAAGGATATGGTTAGAATTAACCTAGATTCATCATTTGTACCACCTGGTGGAATCAAAGTAGTGCATCCACTCTTACAGCCCAAGGCAGAGAGTGGCACTTGGGCACCTGATCAGAAACTCTGCAAATGGAAGCAAGGTAACTGCAGAGGCAGCACGTGTGGTCAAGATTCAAAGTCCTATTCATGCACGTGTGCAGAGGGATATACTTTAAGCCAAGATGGAAAATACTGTGAAGATGTCAATGAATGTGCCTTTTGGAATCATGGCTGTACTCTTGGGTGTGAAAACATCCCTGGATCTTATTATTGCACATGCCCTGTAGGATTTATTCTGCTTCCTGATGGGAAACGGTGTCATCAATTAATTGCCTGTCCGAGCAATACATCTAAATGTAGCCATGACTGTGTTCTGACATCAGATGGTCCCATATGTTTCTGTCCTGAAGGCTCAGTGCTTGAGGCAGATGGAAAAACATGTAGTGGCTGTTCATCACCTGATAATGGTGGATGTAGCCAGCTCTGCCTCCCTCTCAGCCCAGTATCCTGGGAATGTGGTTGCTTTCCTGGGTATGACCTACAACTGGACAAACAAAGCTGTGCAGCATCAGGACCACAACCATTTTTGCTGTTTGCCAATTCTCAAGATATTCGACACATGCATTTTGATGGAACAGATTATGGAACCCTGCTCAGCCAGCAAATGGGAATGGTTTTTGCCCTTGATCACGACCCTGTGGAAAATAAGATATACTTTGCCCATACAGCCCTGAAGTGGATAGAGAGAGCTAACATGGATGGTTCCCAGCGAGAAAGGCTTATTGAGGAAGGAGTGGATGTGCCAGAAGGTCTTGCCATAGATTGGATTGACCGTAAATTCTACTGGACAGACAGCGGAAAATCTCTTATTGAAGGGAGTGATTTAAATGGAAAACATCGTGAGGTAATCATCAAGGAAGACATCTCTCAGCCACGAGGAATTGCTGTTCATCCAATGGCCAAGAGATTATTCTGGACTGATATGGGGATTAATCCACGAATTGAAAGTTCTTCCCTTCAAGGCATTGGCCGACTGGTTATAGCTAGCTCTGATCTGGTCTGGCCCAGTGGAATAACGATTGATTATGTAACTGACAAATTGTACTGGTGTGATACCAAGCTGTCTGTGATTGAGATGGCCAATCTGGATGGTTCAAAACGCCAAAGACTTGCCCAGAACGATGTAGGTCACCCATTTGCTATGGCCGTGTTTGAGGATCACGTGTGGTTCTCTGATTGGACTATGCCATCAATAATAAGAGTGGACAAGAGGACTGGCAAAAACAGGGTACGTCTCCGAGGCAGCATGCTGAAGCCTTCATCACTGGTTGTAGTTCATCCATTGGCAAAACCAGGAGCACAGCCCTGCTTATATCAAAATGGAGGCTGTGAACATATTTGCAAAGAGAGGTTTGGAACTGCTCAATGTTTGTGTCGTGAAGGTTTTGTGAAAGCCCCAGATGGGAAAATGTGTCTGGCTCTGAATGGCCATCAGATACCGGCAGTAGGTAGTGAAGCAGATCTAAGTAATCACGTAACGCCAGGGGATGTCTTACCCAGAAGTGAAGGATTTGAAGATAACATTACAGAATCTCAGCATATGCTAGTGGCCGAAATCATGGTGTCAGATGCTGACGACTGTGCTCCTGTGGGATGCAGTACATGGGCTGAGTGTGTTTCAGAGGGAGAAAATGCCACATGTCAGTGTTTGAAAGGATTTACTGGGGATGGAAAGCTATGTTTTGACATAGATGAATGTGAGATGGGCATCACGATTTGCCCTCCTACCTCCTCAAAGTGCGTCAATACTGAAGGTGGTTATGTTTGTCAGTGCTCAGAAGGCTACCGAGGCGATGGGATCCACTGTCTGGATATTAATGAGTGCCAACTGGGCATGCACACCTGTGGGGAAAATGCCACCTGTACAAATATGGAGGGAAACTATACCTGCATGTGCGCTGGCAGCCTGTCTGAACCTGGACAGATATGTGCTGACTCTACTCCGCCTTCTCATCCCATGGAGGACAGTCACTATTCTGTGAGAAATGGTTATCGGGAATGCCCCTCATCCTATGATGGGTACTGCCTCTATAATGGTGTGTGTATGTACATTGAAGCAGTCGACAGATACGCATGCAACTGTGTTTTTGGCTACGTCGGGGAGCGATGTCAGCACCGAGACCTGAAATGGGAACTGCGCCACGCGGGCCAGGGCCGGCAGCGGCAGGTCGCCGCGGTGGCCGTGGGCGTGGCCGTGCTCGTCCTGCTGCTGCTGCTCGGGCTGGGGGGCGCGCACTGCTACAGGACTAAGAAGTTGTCATCAAAAAATTTAAAGAATCCTTATGAAGAGCCAAGCAGAGAGGGTAGCAGTAGCAGGCCTTCAGACAGCGAGGCTAGGATGGCCTCTTTTCCCCAACCTTGGTTTGTGGTTATAAAGGAACATCAAAATCTCAGGAATGGAAGTCAACCTATGGCCCTCAAGGATGGTGAGTCAGCAGATGTTAGCCAATTTTCCTCTCCAGAGCCAGGGTCAGTAAAACGGACCTCATGGAGAAATGAACACCAGTTATATAAGGACACAGAGCAAGGCTGCTGCACTCCACCATCCAGTAATAGAGGCACCGGCTCTCAGTCAATGGAGCAGAGCTTTTCTGTCCCCTCCTATGAGGCACAGCCCATTGCTTTGGGGGTTGAGAAGCCACAGTCTCTCCTATCAGCTAACCCTTATTGCAACAAAGGGCCCCAGATCCACCACACCAAATGA

Canine HBEGF

ATGAAGCTGCTGCGGTCAGTGGTGCTGAAGCTCTTTCTGGCTGCAGTGCTCTCGGCGTCGGTGACTGGCGAGAGCTTGGGGCGTCTTCGGAGAGGGCTGGCGGCCGGAACTGGCAACCCGGACTCTCCCACCGAATCCACGGACCGGCTGCTGCCCCCGGAAGGCGGCCGGGCCAGGGAAGTCCTGGACTTAGAAGAGACGGACCTGGACCTTTTAAGAGCAGCTGCTTTCTCCTCCAAGCCACAGGCTCTGGCCACACCCAGTAAGGAGGAACGTGGGAAAAAAAAGAAGAAAGGCAAAGGCTTAGGGAGGAAGAGAGACCCGTGTCTTCGGAAATACAAGGACTTCTGCATCCATGGAGAATGCAAATATGTGAAGGAGCTCCGGGCTCCATCCTGCATCTGCCACCCCGGTTACCATGGAGAGAGGTGCCATGGGCTGAGCCTTCCAGTAGAAAATCGCTTATATACTTACGACCATACAACCATCTTGGCTGTGGTGGCTGTGGTGCTGTCATCTGTCTGTCTGCTTGTCATCGTGGGGCTTCTCATGTTTAGGTACCATAGGAGAGGAGGTTATGATGTGGAAAGTGAAGAGAAAGTGAAGTTAGGCATGACTACTTCCCACTGA

Canine EREG

ATGGAGCCGCGCCGCCTGCTGCTGTGCCTGGGTTTCCATCTTCTCCACGCGGTTCTCAGCACCACTGTGATTCCTTCCTGCATGCCGGGAGAATCCGAAGATAATTGCACGGCATTAGTTCAGATAGAAGACAACCCACGTGTGGCTCAAGTGTCAATAATAAAGTGTGGCTCTGACATGAATGGCTACTGTTTGCATGGACAATGCATCTACCTGGTGGACATGAGTCAAACGTACTGCAGGTGTGAAGTGGGTTACACTGGTGTCCGATGCGAGCACTTCTATTTAACTGTCCAACAGCCCTTGAGCAAAGAATATGTGGCTTTGACTGTGATTCTCATTATCTTGTTTCTTATCATAGTCGCCGGTTCCCTATACTACTTCTGCAGATGGTACAGAAATCGAAAAAGTAAAGAACCAAAGCAGGAATACAAAAGGGTGACGTCAGGGGATCCAGCATTGCCACAAGTCTGA

Canine TGFα

ATGGTGCCCTCGGCCGGACGGCTCGCCCTGCTCGCGCTGGGTGTCCTGCTGGCCGCGGGCCAGGCCCTGGAGAACAGCACGTCCGCCCTGAGTGCCAGACCACCAGTGGCCGCTGCAGTAGTGTCTCATTTTAACGATTGCCCAGATTCCCATAGCCAGTTCTGCTTCCACGGGACTTGCAGATTTCTGGTTCAAGAAGATAAACCCGCTTGTGTATGCCACAGTGGCTATGTCGGGGCACGCTGTGAACACGCCGACCTTCTTGCTGTCGTAGCAGCGTCCCAAAAGAAGCAGGCCATCACTGCTCTGGTGGTGGTCTCTATCGTGGCGTTGGCCGTGCTGATTATCGCATGTGTTCTGATCCATTGCTGTCAGGTGCGCAAGCACTGTGAGTGGTGTCAGGCCCTCTTGTGTCGGCATGAGAAGCCCTCCGCATTGCTTAAGGGACGAGCTGCTTGCTGTCATAGCGAGACAGCCGTCTGA

Canine NRG1

ATGGAACCCGACGCCAACAGCAGCAGTAGAGCCCCTGCTGCCTTTCGGGCTAGCTTCCCACCTCTGGAAACCGGCCGGAACCTGAAGAAAGAGGTGTCCAGAGTCCTGTGCAAGAGATGCGCCCTGCCTCCTCGGCTGAAAGAGATGAGAAGCCAAGAGTCTGCCGCCGGAAGCAAGCTGGTGCTGAGATGTGAAACCAGCAGCGAGTACAGCAGCCTGAAGTTCAAGTGGTTCAAGAACGGCAACGAGCTGAACCGGAAGAACAAGCCCCAGAACATCAAGATCCAGAAGAAGCCCGGCAAGAGCGAGCTGAGAATCAGCAAAGCCAGCCTGGCCGATAGCGGCGAGTACATGTGTAAAGTGACCTCCAAGCTGGGCAACGACAGCGCCAGCGCCAATATCACCATCGTGGACAGCAACGACATCATCACCGGCATGCCTGCCAGCACCGAGAGGGCTTATGTGTCTAGCGAGAGCCCCATCCGGATCAGCGTTAGCACAGAAGGCGCCAACACAAGCAGCAGCACCTCTACCAGCACCACCGGCACATCTCACCTGGTCAAGTGCGCCGAGAAAGAAAAGACCTTCTGCGTGAACGGCGGCGAGTGCTTCATGGTCAAGGACCTGAGCAACCCCAGCCGGTATCTGTGCAAGTGTCAGCCCGGTTTTACCGGCGCCAGATGCACCGAGAATGTGCCCATGAAGGTGCAGAATCAAGAGAAGGCCGAGGAACTGTACCAGAAACGGGTGCTGACCATCACAGGCATCTGTATCGCCCTGCTGGTCGTGGGCATTATGTGCGTGGTGGCCTACTGCAAGACCAAGAAGCAGCGGAAGAAGCTGCACGACCGGCTGAGACAGAGCCTGAGAAGCGAGAGAAACAACATGGTCAATATCGCCAACGGACCCCACCATCCTAATCCTCCACCTGAGAACGTGCAGCTGGTCAACCAGTACGTGTCCAAGAACGTGATCAGCTCCGAGCACATCGTGGAACGCGAGGCCGAGACAAGCTTTAGCACCAGCCACTACACCAGCACAGCCCACCACAGCACCACAGTGACACAGACCCCAAGCCACAGCTGGTCCAATGGCCACACCGAGTCCATCATCAGCGAGTCCCACAGCGTGATCATGATGAGCAGCGTGGAAAACAGCCGGCACTCTAGCCCTAGCGGAGGACCTAGAGGCAGACTGAATGGCCTCGGCGGACCCAGAGAGTGCAACAGCTTTCTGAGACACGCCAGAGAGACACCCGACAGCTACAGAGATAGCCCTCACAGCGAGAGATACGTGTCCGCCATGACAACCCCTGCCAGAATGAGCCCCGTGGACTTTCACACACCTAGCAGCCCTAAGAGCCCTCCTAGCGAGACATCCCCTCCAGTGTCTAGCACAACCGTGTCCATGCCTAGCATGGCCGTGTCACCCTTCGTGGAAGAGGAAAGACCTCTGCTGCTGGTCACCCCTCCTAGACTGAGAGAGAAGTACGATCACCACAGCCAGCAGTTCAACAGCTACCACCACAATCCTGCTCACGAGAGCAACAGCCTGCCTCCATCTCCTCTGAGAATCGTCGAGGACGAGGAATACGAGACAACCCAAGAGTACGAGCCCGCTCAAGAGCCCGTGAAGAAACTGACCAGCTCCAGACGGGCCAAGCGGACCAAGCCTAATGGACACATTGCCAACCGGCTGGAAATGGACTCCAATGCCTCTGCCGAGGGCACCAACAGCGAAAGCGAGACAGAGGATGAGAGAGTGGGCGAAGATACCCCATTCCTGGGCATCCAGAATCCTCTGGCCGCCTCTCTTGAAGCCGCTCCTGCTTTTAGACTGGCCGACAGCAGAACAAACCCAGCCGGCAGATTCAGCACCCAAGAGGAACTGCAGGCCAGACTGTCTAGCGTGATCGCCAACCAGGATCCTATCGCCGTCTGA
